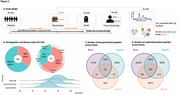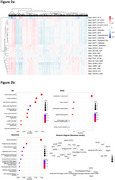# Brain‐Peripheral Proteome Crosstalk in Alzheimer's Disease with and without Diabetes Mellitus

**DOI:** 10.1002/alz70857_102725

**Published:** 2025-12-25

**Authors:** Anat Yaskolka Meir, Ana W. Capuano, Xingyan Wang, Shinya Tasaki, Vishal Sarsani, David A. A. Bennett, Vladislav A Petyuk, Liming Liang, Zoe Arvanitakis

**Affiliations:** ^1^ Harvard T.H. Chan School of Public Health, boston, MA, USA; ^2^ Rush Alzheimer's Disease Center, Rush University Medical Center, Chicago, IL, USA; ^3^ Rush University Medical Center, Chicago, IL, USA; ^4^ Broad Institute, Cambridge, MA, USA; ^5^ Pacific Northwest National Laboratory, Richland, WA, USA; ^6^ Harvard T.H. Chan School of Public health, Boston, MA, USA

## Abstract

**Background:**

Previous studies showed alterations in protein expression in the brain tissue of individuals with Alzheimer's disease (AD). However, despite a link between AD dementia and diabetes mellitus (DM), little to no studies examined cross‐tissue proteome to detect central‐peripheral associations and the link with AD with/without DM.

**Method:**

We used data from the Rush Memory and Aging Project (MAP). AD status was determined using the NIA‐AA pathology criteria. Participants were matched by DM status (self‐reported medical history and/or the use of anti‐diabetic medications). TMT‐based Phosphoproteome profiling in postmortem human brain prefrontal cortex (*N* = 191) and deltoid muscle (*N* = 191) tissues and antemortem serum samples (*N* = 96) of older adults were measured.

**Result:**

The mean (SD) death age was 89.9 (5.7), 65% were women, 64% met pathologic criteria for AD, and 50% had DM. 1,655 and 5,181 annotated phosphorylated and unphosphorylated peptides, respectively, overlapped between the brain and muscle tissues (‐0.461<r<0.824, 52,412 phosphorylated pairs with FDR<0.05; ‐0.481<r<0.855, 2,076 unphosphorylated pairs with FDR<0.05) (Figure 1). 61 and 452 peptides overlapped between brain and serum (‐0.345<r< 0.460, one phosphorylated pair with FDR<0.05; ‐0.444<r<0.799, unphosphorylated 1,445 pairs with FDR<0.05). 779 muscle phosphorylated peptides were significantly correlated with a set of 23 previously uncovered pathological AD‐related brain phosphorylated peptides (FDR<0.05) (Figure 2a). Gene set enrichment analysis of these muscle phosphorylated peptides revealed multiple pathways, including muscle contraction and neurodegenerative diseases (Figure 2b). Overall, muscle phosphorylated peptides were enriched with lower *p*‐values in our multivariable (MV) models for pathological AD association, including within DM strata. Muscle SARS2‐S126, correlated with three pathological AD‐related brain phosphorylated peptides, was differentially expressed in pathological AD (FDR=0.011; MV models). In a set of peptides related to insulin resistance (IR) pathways, serum phosphorylated peptides were enriched with lower *p*‐values in our MV models examining pathological AD.

**Conclusion:**

Overall, muscle peptides were mostly related to pathological AD, while serum peptides were more likely to be related to IR in AD. The crosstalk between central and peripheral peptide expression may be the first step in uncovering AD markers in distant tissues.